# Negative Pressure Waves Based High Resolution Leakage Localization Method Using Piezoceramic Transducers and Multiple Temporal Convolutions

**DOI:** 10.3390/s19091990

**Published:** 2019-04-28

**Authors:** Guangmin Zhang, Siu Chun Michael Ho, Linsheng Huo, Junxiao Zhu

**Affiliations:** 1 School of Electrical Engineering and Intelligentization, Dongguan University of Technology, Dongguan 523808, China; zhanggm@dgut.edu.cn; 2 Department of Mechanical Engineering, University of Houston, Houston, TX 77204, USA; smho@uh.edu; 3 State Key Laboratory of Coastal and Offshore Engineering, Dalian University of Technology, Dalian 116024, China

**Keywords:** negative pressure wave (NPW), leakage detection, leakage localization, piezoceramic sensor, multiple temporal convolutions

## Abstract

The negative pressure wave (NPW) signals generated by a pipeline leakage often have a long signal duration. When these signals are utilized to compute the leakage position, the long signal duration will result in a large area being considered as leakage area. The localization resolution is low. A novel high-resolution localization algorithm is developed for pipeline leakage detection using piezoceramic transducers in this paper. The proposed algorithm utilizes multiple temporal convolutions to decrease the localization functional values at the points close to the leakage, in order to reduce the range of the leakage area revealed by the proposed algorithm. As a result, the localization resolution is improved. A measured experiment was conducted to study the proposed algorithm. In the experiment, the proposed algorithm was used to monitor a 55.8 m pressurized pipeline with two controllable valves and two Lead Zirconate Titanate (PZT) sensors. With the aid of the piezoceramic sensor, the experimental results show that the proposed algorithm results in a resolution which is better than that of the traditional method.

## 1. Introduction 

As a multi-disciplinary research field [1,2,3,4], structural health monitoring (SHM) is applied in multiple areas [5,6,7]. SHM technology can evaluate the condition of structural health and, through appropriate data processing and interpretation, issue early warning, if needed. Recently, structural health monitoring with Lead Zirconate Titanate (PZT) transducer has gained extensive attraction [8,9]. PZT transducers can exchange energy between mechanical energy and electrical energy [10,11], and therefore, they can work as an actuator to general stress or ultrasonic waves or a sensor to detect such waves [12,13,14,15]. PZTs are often used to build vibrations sensors, such as accelerometers, for vibration detection and control [16,17], as well as ultrasonic transducers [18,19,20] and acoustic emission probes [21,22,23] for SHM. Furthermore, this type of transducer can be bonded onto a structural surface or embedded in a concrete or composite structure during the structural fabrication process. 

The SHM with PZT transducer can be performed by using several methods. One method is based on wave propagation [24]. Usually, two PZT transducers are integrated with the monitoring structures: one works as the actuator, and the other works as the receiver. By analyzing the energy change of the received signal, the health status of the structures can be evaluated. A number of SHM investigations have been adopted that have a monitoring scheme, such as the bolt connection status and bolt loosening monitoring [25,26,27], the damage evaluation [28,29,30] and detection [31,32], bonding status monitoring of composite structures [33,34,35,36], grouting compactness monitoring [37,38,39]. Furthermore, to obtain the detail of the structural damage, some investigators developed imaging algorithms which process the wave signals captured by a PZT transducer array to reveal damages of the structures [40,41,42,43]. In addition to the active SHM based on PZT, the PZT transducers are also employed for passive monitoring. For example, PZT sensor arrays were used to detect and localize the impact on various structures [44,45]. Another type of PZT application is based on electromechanical impedance (EMI) [46,47,48]. EMI is widely used for the health monitoring of various structures, for example, strength development monitoring of cementitious material [49], cable force monitoring in tendon-anchorage [50], bolt-joint structural health monitoring [51], impedance monitoring in tendon-anchorage [52], and local strand-breakage detection in multi-strand anchorage system [53]. Furthermore, the performance of EMI based SHM method can be improved by using multilevel wavelet decomposition [54], metamaterial plasmons [55], artificial neural networks [56] and fuzzy network [57], locally resonant piezoelectric metastructure [58] and so on.

As an important part of infrastructure, pipelines play an important role in a nation’s economy. However, pipelines are subjected to adverse factors, such as impacts and corrosion [59,60,61,62], which may lead to leakage. Since leakages in pipelines can cause serious accidents, PZT transducers have been widely used for pipeline leakage detection to provide early warning [63,64,65,66], taking their advantages of wide bandwidth and dual actuating and sensing capacities. When a leakage occurs, the negative pressure wave (NPW) [67], which is generated by the leak, propagates toward both sides of the pipeline and is captured by PZT transducers. By analyzing the NPW signals, the leakage’s location can be revealed. For instance, Zhao et al. [68] developed a wavelet analysis based leak localization method of the natural gas pipeline. Hu et al. [69] developed a harmonic wavelet-based pipeline small leakage detection method with noise suppression. Liu et al. [70] proposed a new leak detection and location method based on the propagation law of leakage acoustic waves. 

Time reversal (TR) can make the signals focus on the signal source by the physical method [71,72,73] or by computation [74,75,76,77]. Therefore, a lot of localization methods based on time reversal are developed. Zhao et al. proposed a cross-sectional scanning-based time reversal defect localization method [74]. Zheng et al. developed a unique location-specified signature based high accuracy TR localization approach [75]. Qiu et al. propose a TR localization method which can obtain the impact region image by processing the signals estimated with using the digital sequences [76]. However, when the time reversal method is applied to localize impact region or leakage area, due to the long duration of the impact signal and the leakage signal, the localization method often has a low localization resolution [76,77]. 

In this paper, a new high-resolution localization algorithm for pipeline leakage detection using PZT transducers and multiple temporal convolutions is developed. The proposed algorithm is based on the temporal characteristic of the back-propagation signal. With using multiple temporal convolutions, the localization functional values at the points close to the leakage drop dramatically. Therefore, the size of the leakage area revealed by the proposed algorithm gets small, and the localization resolution can be improved. The proposed algorithm was employed for the pipeline leakage localization and a measured experiment was executed. The results show that the proposed algorithm can give a good idea of the two leakage positions in a PVC pipeline with 55.8 m length. Moreover, the novel localization algorithm offers a resolution of about 2.5 m which cannot be obtained by using the traditional localization algorithm.

## 2. Theory of the Proposed Algorithm

**Figure 1:**
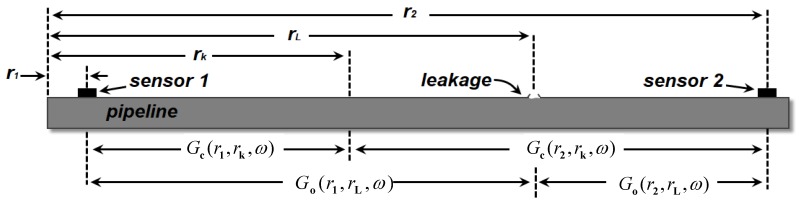
Model of the proposed algorithm.

For corresponding to the pipeline leakage experiment, a model of pipeline is built to describe the proposed algorithm. For a gas pipeline, an NPW generated by a leakage will propagate from the leakage location to the two ends of the pipeline. Assume that two PZT transducers are used to catch the NPW, and the *n^th^* sensor locates at $r_{n}$, as shown in Figure 1. A leakage happens at $r_{L}$. Assume the time of NPW occurrence is *T**_L_***. All sensors work synchronously. The Fourier transform of the leakage signal captured by the $n^{th}$ sensor can be represented as:

(1) $$Y(\omega ,r_{n},r_{L})=G_{o}(r_{n},r_{L},\omega )X(\omega )e^{-i\omega T_{L}}$$

where X(ω) is the Fourier transform of x(t), and $G_{o}(r_{L},r_{n},\omega )$ is the measured transfer function representative of propagation from the leakage to the $n^{th}$ sensor. The subscript “o” emphasizes that this is obtained by the experimental approach.

First reverse the NPW signal in the time domain. Since inverse a signal in time domain is equivalent to taking the complex conjugate in frequency domain, the inversion version of Equation (1) can be represented as:

(2) $$Y_{INV}(\omega ,r_{n},r_{L})=G_{o}{}^{*}(r_{n},r_{L},\omega )X^{*}(\omega )e^{i\omega T_{L}}$$

where “*” represents the complex conjugate.

Then, the signal $Y_{INV}(\omega ,r_{n},r_{L})$ back-propagates at the $n^{th}$ sensor’s position. In the proposed algorithm, the back-propagation is realized via convoluting with $G_{c}(r_{n},r_{k},\omega )$, where $G_{c}(r_{n},r_{k},\omega )$ is the computational transfer function representing the “propagator” from location $r_{n}$ to generic observation point $r_{k}$. The symbol “c” emphasizes that this is computed in software. At the generic observation point $r_{k}$ of the monitoring domain, the back-propagation signal of the $n^{th}$ sensor is illustrated as:

(3) $$F_{n}^{}(\omega ,r_{k},r_{n})=G_{o}{}^{*}(r_{n},r_{L},\omega )G_{c}(r_{n},r_{k},\omega )X^{*}(\omega )e^{i\omega T_{L}}$$

Due to the reciprocity of NPW, we can assume that the computational transfer function matches the measured data, namely $G_{o}(r_{n},r_{L},\omega )=G_{c}(r_{n},r_{L},\omega )$. All back-propagation signals will focus at the leakage position $\left(r_{k}=r_{L}\right)$ at the time t = −*T_L_*. To cancel *T_L_*, the signals will be processed as following. $f_{1}^{}(t,r_{k},r_{1})$ is inversed in time domain and then convolved with $f_{2}^{}(t,r_{k},r_{2})$. The corresponding output signal can be represented by:

(4) $$\begin{array}{ll} q(r_{k},r_{1},r_{2},t) & =f_{1}^{}(-t,r_{k},r_{1})⊗f_{2}^{}(t,r_{k},r_{2})\\ & =\frac{1}{2\pi }\int_{}^{}F_{2}^{}(\omega ,r_{k},r_{2})F_{1}^{*}(\omega ,r_{k},r_{1})e^{i\omega t}d\omega \end{array}$$

where $F_{1}^{}(\omega ,r_{k},r_{1})$ is the frequency domain expression of $f_{1}^{}(t,r_{k},r_{1})$, $F_{2}^{}(\omega ,r_{k},r_{2})$ is the frequency domain expression of $f_{2}^{}(t,r_{k},r_{2})$, “⊗” represents the convolution operation.

$q(r_{k},r_{1},r_{2},t)$ is symmetric with respect to the reference time t = 0 at $r_{k}=r_{L}$, neither is at other position. For using the focal characteristic of $q(r_{k},r_{1},r_{2},t)$, self-convolution for $q(r_{k},r_{1},r_{2},t)$ is carried out as follows:

(5) $$s(r_{k},r_{1},r_{2},t)=q(r_{k},r_{1},r_{2},t)⊗q(r_{k},r_{1},r_{2},t)$$

Design the localization functional as:

(6) $${I_{p}(r_{k})=s(r_{k},r_{1},r_{2},t)|}_{t=0}$$

The localization functional value of the proposed algorithm at the leakage position is:

(7) $$I_{p}(r_{L})=\frac{1}{2\pi }\int_{}^{}{\left|X(\omega )\right|}^{4}{\left|G_{o}(r_{2},r_{L},\omega )\right|}^{4}{\left|G_{o}(r_{1},r_{L},\omega )\right|}^{4}e^{i\omega t}d\omega$$

**Figure 2:**
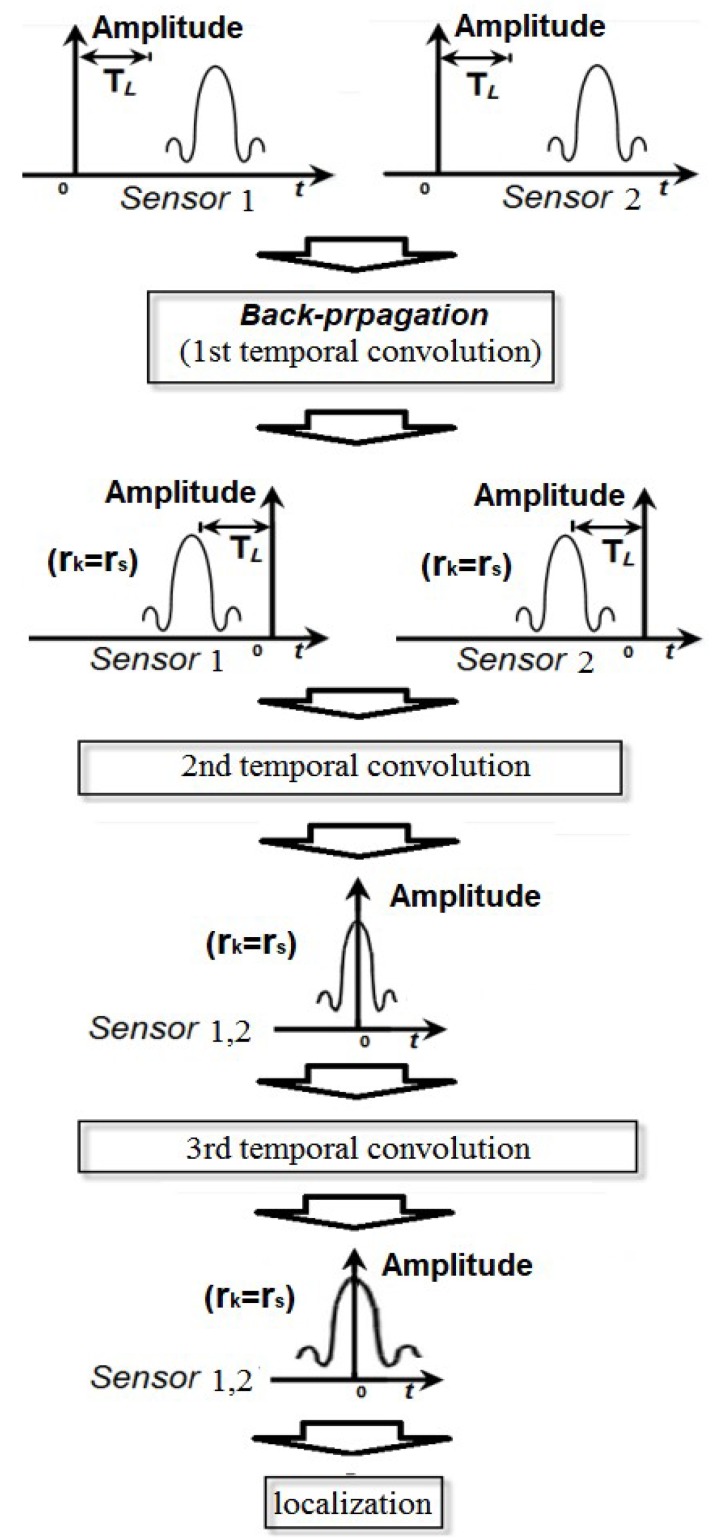
The flow diagram of the proposed algorithm.

The flow diagram of the proposed algorithm is shown in Figure 2.

## 3. Analysis About Resolution Improvement 

For pipeline leakage, the localization functional of the traditional time reversal localization algorithm based on the maximum value [77] is represented as follows:

(8) $$I_{CTR}(r_{k})=Max\sum_{n=1}^{N}f_{n}(t,r_{k},\;r_{n})$$

where $f_{n}(t,r_{k},\;r_{n})$ is the back-propagation temporal signal of the $n^{th}$ sensor.

The localization functional value of the traditional time reversal localization algorithm at the leakage position is:

(9) $$I_{CTR}(r_{L})=\frac{1}{2\pi }\int_{}^{}X^{*}(\omega )\left({\left|G_{o}(r_{2},r_{L},\omega )\right|}^{2}+{\left|G_{o}(r_{1},r_{L},\omega )\right|}^{2}\right)e^{i\omega t}d\omega$$

Take into account a generic observation point $r_{z}$ on the pipeline, and then, the computational transfer function at $r_{z}$ can be represented as:

(10) $$G_{c}(r_{n},r_{z},\omega )=G_{c}(r_{n},r_{L},\omega )e^{i\theta_{n,L,z}}$$

where $\theta_{n,L,z}$ is the phase difference between $G_{c}(r_{n},r_{z},\omega )$ and $G_{c}(r_{n},r_{L},\omega )$.

According to Equation (8), the localization functional value of the traditional time reversal localization algorithm at $r_{z}$ can be represented as:

(11) $$I_{CTR}(r_{z})=\frac{1}{2\pi }\int_{}^{}X^{*}(\omega )\left({\left|G_{o}(r_{2},r_{L},\omega )\right|}^{2}e^{i\theta_{2,L,z}}+{\left|G_{o}(r_{1},r_{L},\omega )\right|}^{2}e^{i\theta_{1,L,z}}\right)e^{i\omega t}d\omega$$

The localization functional value of the proposed algorithm at $r_{z}$ can be written as:

(12) $$I_{p}(r_{z})=\frac{1}{2\pi }\int_{}^{}{\left|X(\omega )\right|}^{4}{\left|G_{o}(r_{2},r_{L},\omega )\right|}^{4}{\left|G_{o}(r_{1},r_{L},\omega )\right|}^{4}e^{i2*(\theta_{2,L,z}+\theta_{1,L,z})}e^{i\omega t}d\omega$$

where the point $r_{z}$ is very close to the leak point, namely $r_{z}\approx r_{L}$, $\theta_{n,L,z}$ approaches to zero, $I_{CTR}(r_{z})\approx I_{CTR}(r_{L})$. The generic observation point $r_{z}$ will be classified as the leakage area. However, due to the superposition of various phase difference and the phase difference amplification of two times, the phase difference in Equation (12) enlarges apparently, $I_{p}(r_{z})$ decreases. Due to the decrease of the localization functional value at the point close to the leakage, the number of the points considered as the leakage area declines, the leakage area given by the proposed algorithm gets small. Therefore, the localization resolution is improved.

## 4. Experimental Result and Discussion

**Figure 3:**
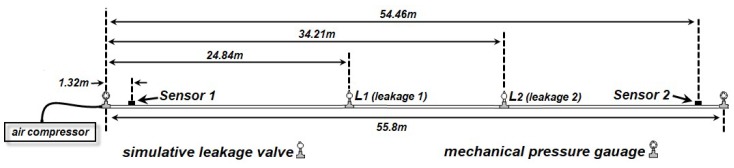
Schematic diagram of the measured experiment.

As shown in Figure 3, a pipeline with 55.8 m length was composed of five 0.2 m sections, six 9.1 m straight sections and ten 90° elbow connectors. Two PZT sensors with size of 15 mm × 10 mm × 0.4 mm were located at 1.52 m and 54.08 m respectively. The Two PZT sensors were mounted on the outer surfaces of the pipeline by using the super glue [78]. Two valves located respectively at 24.84 m and 34.21 m are used to produce leakage signals. The special locations of the leakages and the sensors are listed in Table 1. The PZT is APC850 whose properties are available from the manufacturer [79]. The parameters of APC850 are shown in Table 2. The data acquisition system is a NI PXI-5105 Digitizer. The equipment was triggered by the voltage signal of the sensor 1 with a −0.02 V trigger level. The sampling rate of the experiment is 100 KS/s. A compressor pumped air into the pipeline, and a leakage was produced when a valve on the pipeline is opened. Then, the PZT sensors caught the leakage signals.

The computational transfer function $G_{c}(r_{n},r_{k},\omega )$ is represented as:

(13) $$G_{c}(r_{n},r_{k},\omega )=\frac{\exp(-jkR(r_{n},r_{k}))}{R(r_{n},r_{k})}$$

where $k=\omega /v_{g}$ is the wave number, and $v_{g}$ is the NPW velocity. In this experiment, the velocity is 300 m/s [63]. For the observation point $r_{k}$, we let

(14) $$R(r_{n},r_{k})=\left|r_{k}-r_{n}\right|$$

denote the distance between the $n^{th}$ sensor at $r_{n}$ and the observation point $r_{k}$. 

**Figure 4:**
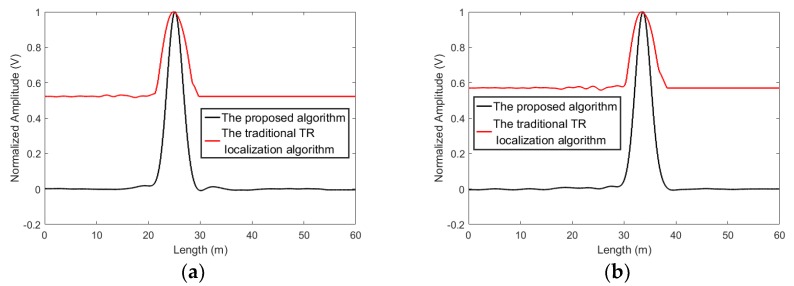
The localization results obtained by using the proposed algorithm and the traditional time reversal localization algorithm based on the maximum of the time reversed signal. (**a**) L1 and (**b**) L2.

The measured data is processed by using the traditional time reversal localization algorithm [77] and the proposed algorithm. It is worthwhile mentioning that the traditional time reversal localization algorithm used the maximum value of time reversed signal to localize the leakage, since the occurrence time of the leakages is unknown. In addition, in the results based on the two methods, the localization functional values are normalized for the purpose of investigating the resolution, as shown in Figure 4. From Figure 4, it can be seen that the traditional time reversal localization algorithm can identify the leakages’ positions. However, since the duration of the signal is long, the time reversed signals still superposed with each other. The localization functional value of the traditional time reversal localization attenuated slowly. Therefore, numbers of points close to the leakages are classified into the leakage area. That means the localization resolution of the traditional time reversal localization algorithm based on the maximum of the time reversed signal declines. On the other hand, the proposed algorithm applies multiple temporal convolutions to increase the phase deference between the signal at the leakage point and the signals at non-leakage points, thus decreasing the localization functional values at the points close to the leakage. That means that fewer points are classified into the leakage area, and the localization resolution is improved.

The resolution is highly related to the question of the maximum size of leakage areas. It sets a boundary limit between points having different signal signatures. Usually, the boundary is chosen at 0.7 (−3 dB) [77]. The points with localization functional values larger than −3 dB will be classified into the leakage area. As shown in Figure 4, for the traditional time reversal localization algorithm, the −3 dB area of the L1 is from length = 22.8 m to length = 27.9 m and the −3 dB area of the L2 ranges from length = 30.8 m to length = 36.5 m. The resolution of the traditional time reversal localization algorithm is unsatisfied. Using the proposed algorithm, the −3dB area of the L1 covers from length = 23.9 m to length = 26.4 m and the −3 dB area of the L2 is from length = 32.5 m to length = 34.9 m. Apparently, the −3 dB areas based on the proposed algorithm are smaller than those based on the traditional time reversal localization algorithm. 

**Figure 5:**
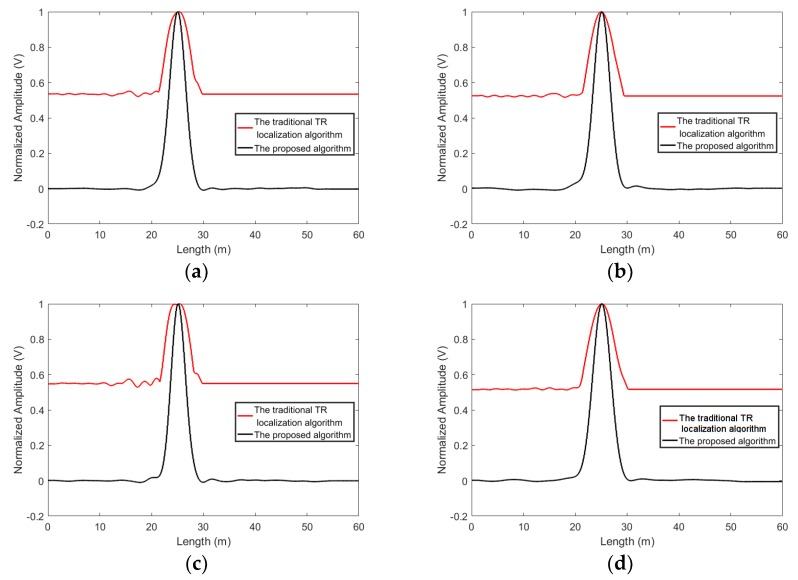
The four extra experiment results obtained by using the proposed algorithm and the traditional time reversal localization algorithm for Leakage 1, (**a**) Data 1, (**b**) Data 2, (**c**) Data 3, (**d**) Data 4.

**Figure 6:**
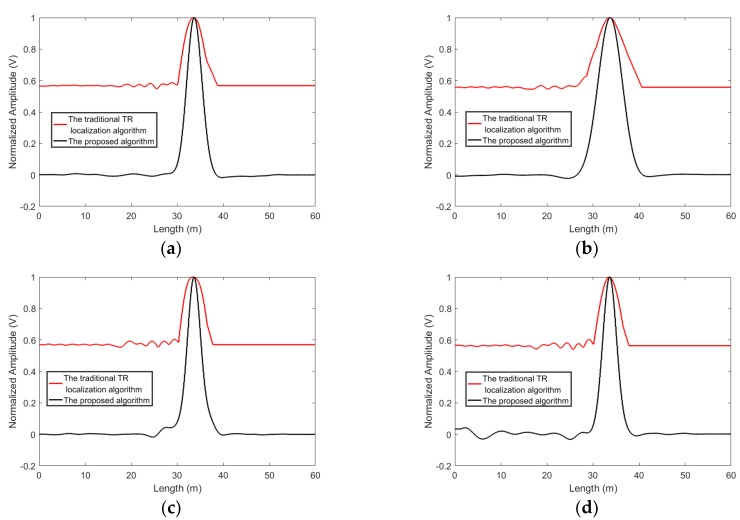
The four extra experiment results obtained by using the proposed algorithm and the traditional time reversal localization algorithm for Leakage 2. (**a**) Data 1, (**b**) Data 2, (**c**) Data 3, (**d**) Data 4.

The four extra measured experiments were conducted to investigate repeatability of the proposed algorithm. As shown in Figure 5 and Figure 6, in the all results, the curves of the proposed algorithm are narrower than those of the traditional time reversal localization algorithm at the leakage areas. This means that the repeatable performance can be achieved by using the proposed algorithm. 

**Figure 7:**
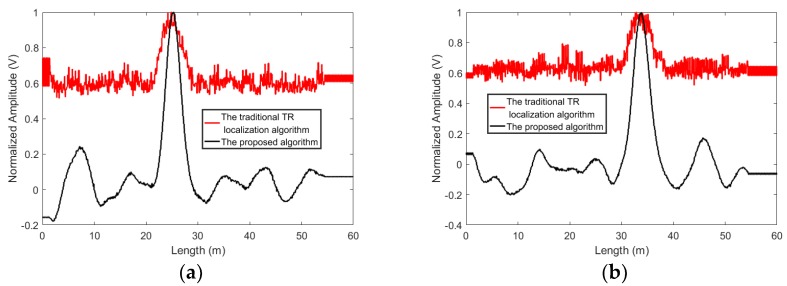
The localization results obtained by using the proposed algorithm and the traditional time reversal localization algorithm at *SNR* = −5dB, (**a**) L1 and (**b**) L2.

**Figure 8:**
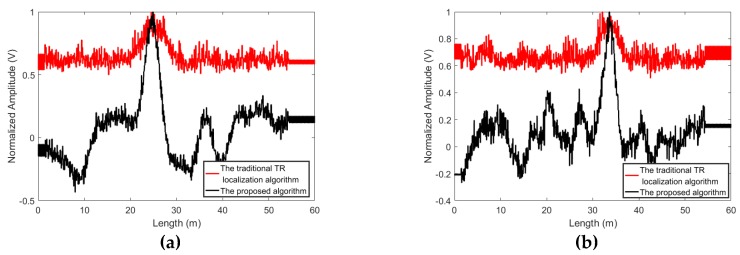
The localization results obtained by using the proposed algorithm and the traditional time reversal localization algorithm at *SNR* = −10dB, (**a**) L1 and (**b**) L2.

The proposed algorithm can better reject the noise. To compare the proposed algorithm with the traditional time reversal localization algorithm and the algorithm presented in [80], experiments are conducted and the standard white Gaussian noise is added to the acquired signals. The results of the proposed algorithm and the traditional time reversal localization algorithm at *SNR* = −5 dB and −10 dB are shown in Figure 7 and Figure 8. When *SNR* = −5 dB, the proposed algorithm can suppress the interference of noise and give a good idea of the leakage’s position. When *SNR* drops to −10 dB, the noise causes the localization curves of the proposed algorithm to distort. However, the leakage locations can still be distinguished. Contrastingly, in the results based on the traditional time reversal localization algorithm, due to the effect of the noise, the red curves are not smooth. It is difficult to identify the exact location of the leakage. 

**Figure 9:**
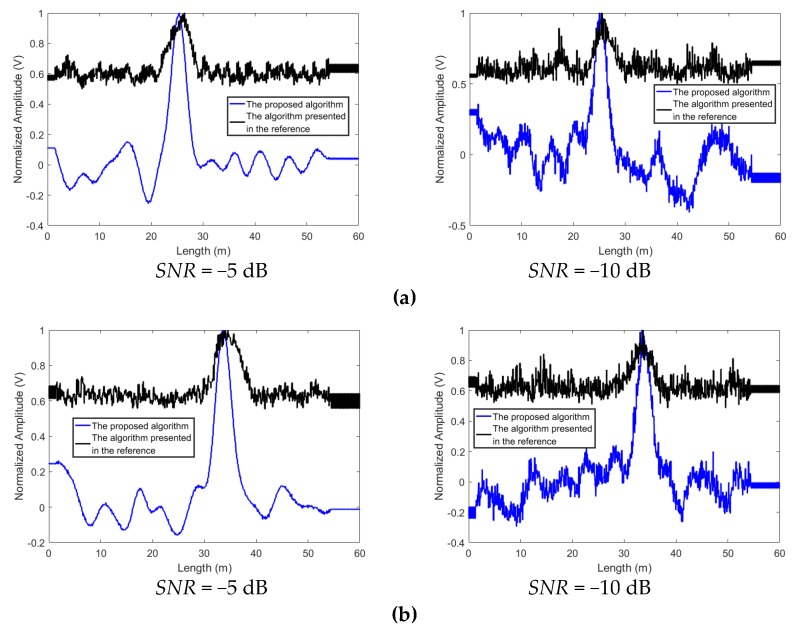
The localization results obtained by using the proposed algorithm and the method (the parameter ***p*** = 1) presented in [80] at various *SNR*s, (**a**) L1 and (**b**) L2.

The focusing signal is symmetric with respect to the reference time t = 0 at the leakage location, neither is the noise. Therefore, the proposed algorithm which localizes the leakage by computing the symmetry of the signals can enhance the focusing signal energy and suppress the noise. On the other hand, since the traditional time reversal localization algorithm identified the leak position by superposing the two acquired signals directly, the NPW signals and the noise can be strengthened synchronously via the traditional time reversal localization algorithm. Therefore, the effect of the noise is more remarkable on the results of the traditional time reversal localization algorithm. Additionally, since the algorithm presented in [80] also localized the leakages by superposing the two signals directly, the same influence of the noise can be seen in Figure 9. Obviously, the proposed method can better reject the noise.

**Figure 10:**
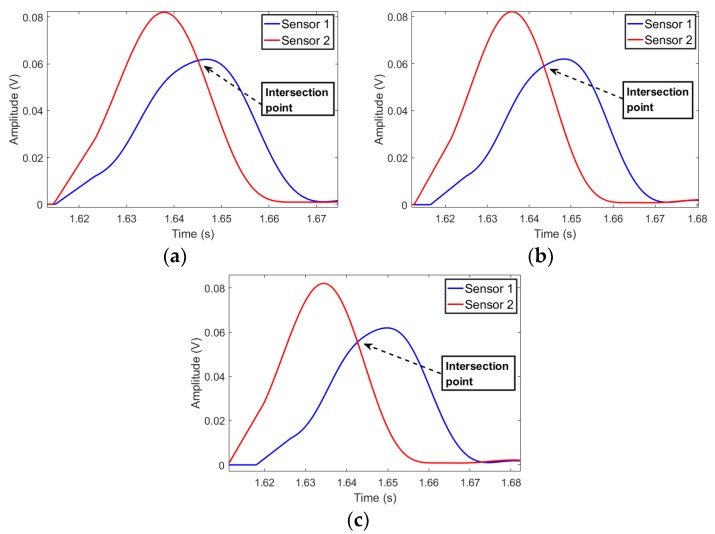
The L2’s time reversed signals, (**a**) 34.71 m, (**b**) 35.21 m, (**c**) 35.71 m.

**Figure 11:**
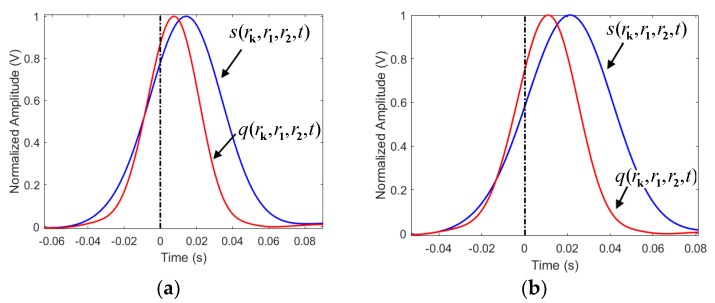

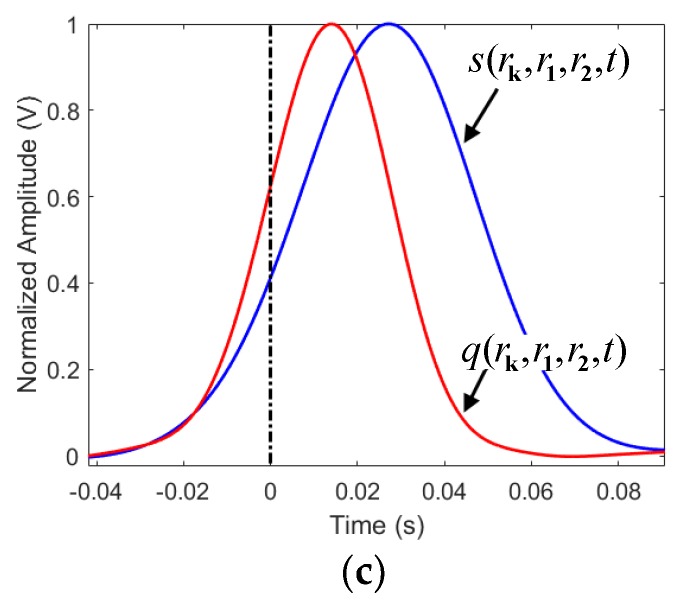
The waveforms of $q(r_{k},r_{1},r_{2},t)$ and $s(r_{k},r_{1},r_{2},t)$ at various $r_{k}$. (**a**) $r_{k}$= 34.71 m, (**b**) $r_{k}$= 35.21 m, (**c**) $r_{k}$= 35.71 m.

The L2’s time reversed signals from the two sensors are shown in Figure 10. As shown in Figure 10, at the observation points, the time reversed signals from the two sensors are slowly far from each other, with the increase of the distance between the observation point and the leakage point. At the point of intersection of two waveform curves, the amplitudes of the signals attenuate slowly. Therefore, the maximum signal amplitude of the traditional time reversal localization algorithm at the points close to the leakage position slowly decreases. The waveforms of $q(r_{k},r_{1},r_{2},t)$ and $s(r_{k},r_{1},r_{2},t)$ at various $r_{k}$ are shown in Figure 11. Since Equation (7) enlarges the phase of the output signal, the waveform of $s(r_{k},r_{1},r_{2},t)$ is further away from the reference time t = 0, as compared to the waveform of $q(r_{k},r_{1},r_{2},t)$. Furthermore, at t = 0, the amplitude of $s(r_{k},r_{1},r_{2},t)$ is lower than that of $q(r_{k},r_{1},r_{2},t)$. Therefore, the localization functional values of the proposed algorithm attenuate fast.

The temperature can affect the acquired signals, and the effect has been investigated [47]. In this experiment, the effect of temperature variation on the performance of the proposed method was not investigated, due to the limitation of the current experiment condition. However, the corresponding investigation will be considered in future work.

## 5. Conclusions

When the conventional localization algorithms are applied for low frequency signal passive detection, such as pipeline leakage detection, their resolutions will be quite low due to the long signal duration. In this paper, a novel localization algorithm is designed to enhance the resolution of pipeline leakage localization using piezoceramic transducers. Based on the temporal characteristic of the time reversal signals, multiple convolution operations were designed and performed. The multiple convolution operations can increase the phase deference between the signal at the leakage point and the signals at non-leakage points, in order to decrease the localization functional values at the points close to the leakage. Therefore, the leakage area revealed by the proposed algorithm gets smaller, and the localization resolution is improved. The proposed localization algorithm was employed in a detection system with PZT sensors for localizing leakages in a pressurized pipeline. The results indicate the proposed algorithm can provide a good localization map of the leakage. Furthermore, the proposed algorithm can obtain a resolution of about 2.5 m, which represents a significant improvement, as compared to those of the traditional one.

## Figures and Tables

**Table 1:** Coordinates of sensors and leakages.

Sensors or Leakages	Length Direction (Unit: m)
Sensor 1	1.32 m
Sensor 2	54.46 m
Leakage 1 (L1)	24.84 m
Leakage 2 (L2)	34.21 m

**Table 2:** Parameters of Lead Zirconate Titanate (PZT) sensor.

Parameters	Relative Dielectric Constant	Electromechanical Coupling Factor	Piezoelectric Charge Constant (10^−12^ C/N or 10^−12^ m/V)	Piezoelectric Voltage Constant (10^−3^ Vm/N or 10^−3^ m^2^/C)
value	1900	0.72 (k33)	400 (d33)	24.8 (g33)
